# Deaths after Operations

**Published:** 1908-06-13

**Authors:** 


### DEATHS AFTER OPERATIONS.

Except in rare cases it is never an easy thing to decide whether a death which has occurred after a surgical operation has been accelerated or not by that operation. If it has been so accelerated the death is unnatural or violent, and comes within section 3 of the Coroners Act of 1887, which makes an inquest imperative in all such cases. Upon this point the coroners of the present day seem to be unanimous; and it must be conceded that an inquest is called for in the case of any death which there is the least reason to suppose has been hastened by an operation that was unnecessary, or was carried out with negligence or want of skill. Such a death is certainly unnatural, and in the public interest it should be inquired into with all care. But these occurrences are happily uncommon. On the other hand, deaths are constantly happening within a short time of operations which have been carefully performed by competent surgeons. Some of these operations are undertaken with the hope of effecting a cure, others merely as palliative measures. In either case, and especially in the latter, the necessity for an inquest is not apparent. Indeed, the holding of a public inquiry into the circumstances of every death occurring within measurable distance of an operation would not only be a sad waste of public time and money, but a very serious set-back to the progress of surgery. Since those who are in professional attendance upon such cases cannot say whether or not death has been appreciably hastened by operative interference, it is hardly reasonable to suppose that a coroner's jury will be able to decide. Nevertheless, Mr. John Troutbeck, at a recent inquest, delivered himself of a speech which clearly shows that he is in favour of an inquest being held in all such cases. It is difficult to see in what way wholesale public inquiries will help to solve such a complex problem. They will certainly frighten the public, and cause grave dislocation of the work of hospital staffs. If an inquest were ordered on every case of death in which an operation had been performed in connection with the last illness, the life of a busy surgeon would become intolerable. We cannot believe that there is urgent need for reform in this matter. The conditions of operative surgery have undoubtedly changed during the past few decades, and operations are much more frequent than they used to be. But the death-rate after operations has steadily diminished; and it would be a serious blow to surgical progress to punish every failure to prolong life by the discomfort and publicity of a coroner's inquest. Sir Victor Horsley, who attended the Battersea Coroner's Court on the occasion in question, to give evidence as to the operation he had performed, said that the case was such an ordinary one that he could not understand why an inquest was held. We imagine that the majority of the profession will agree with Sir Victor Horsley that inquests are only desirable in those cases in which suspicion arises that the operation was unjustifiable or was negligently performed. But Mr. Troutbeck has declared that the matter shall not stop there. A very serious condition of affairs has been revealed to him, and public measures must be taken. Such questions, he maintains, cannot be left to the judgment of any one profession, however honoured or however skilled. We may therefore expect to hear more on this subject before very long. In the meantime we sincerely hope that zeal will not get the better of prudence, and that one who exercises judicial functions will think more than once before he uses the strict letter of the law as a means towards a very doubtful end.

